# Case of convulsive seizure developing during electroretinographic recordings: a case report

**DOI:** 10.1186/s12883-018-1051-2

**Published:** 2018-04-25

**Authors:** Yuko Hayashi, Gen Miura, Akiyuki Uzawa, Takayuki Baba, Shuichi Yamamoto

**Affiliations:** 1 Department of Ophthalmology and Visual Science, Chiba University Graduate School of Medicine, Inohana 1-8-1, Chuo-ku, Chiba, 260-8670 Japan; 2 Department of Neurology, Graduate School of Medicine, Chiba University, Chiba, Japan

**Keywords:** Electroretinogram (ERG), Seizure, Epilepsy, Photosensitivity

## Abstract

**Background:**

To present our findings in a case of convulsive seizures and loss of consciousness that developed during recording electroretinograms (ERG).

**Case presentation:**

A 34-year-old man had reduced vision in his left eye for about 15 years, and night blindness for about two years. His visual acuity was 20/15 in the right eye and 20/50 in the left eye. The fundus was normal but the sensitivity in the macular region of the left eye was decreased. Optical coherence tomography (OCT) showed partial loss of the interdigitation zone. Upon completion of the flicker ERG recording, a paralysis developed in both upper limbs, then convulsions of the lower limbs followed by a loss of consciousness. The convulsions disappeared after an intravenous injection of diazepam. After that incident, he reported that he had had previous conscious-loss seizures.

**Conclusions:**

Photosensitive epileptic seizures can occur with the light stimuli used for conventional ERG recordings. We recommended that clinicians request information on any prior seizure episodes of the patients and their family members before ERG recordings.

## Background

It is well known that an epileptic seizure can be triggered by exposure to intermittent light stimulation. In Japan, many viewers had epileptic seizures while viewing a television animation program, “Pocket Monster”, in 1997. Many individuals visited hospitals because of experiencing seizures after watching a 12 Hz red/cyan blinking image lasting 4 s [1]. Of these cases, 76% were first episodes of seizures, and most were tonic-clonic seizures. A similar phenomenon has been reported not only from viewing TV images but also from viewing video game images, and the guidelines for TV broadcasting in each country have been altered to minimize these potential seizure-eliciting images.

Clinically, ERGs and visual evoked potentials (VEPs) are elicited by intermittent light and pattern-reversal stimuli. However, a search of Medline/PubMed did not extract any publications of convulsive seizures that developed during ERG and VEP recordings.

Thus, the purpose of this report is to present our findings in a case of convulsive seizures and loss of consciousness that developed during ERG recordings.

## Case presentation

**Fig. 1:**
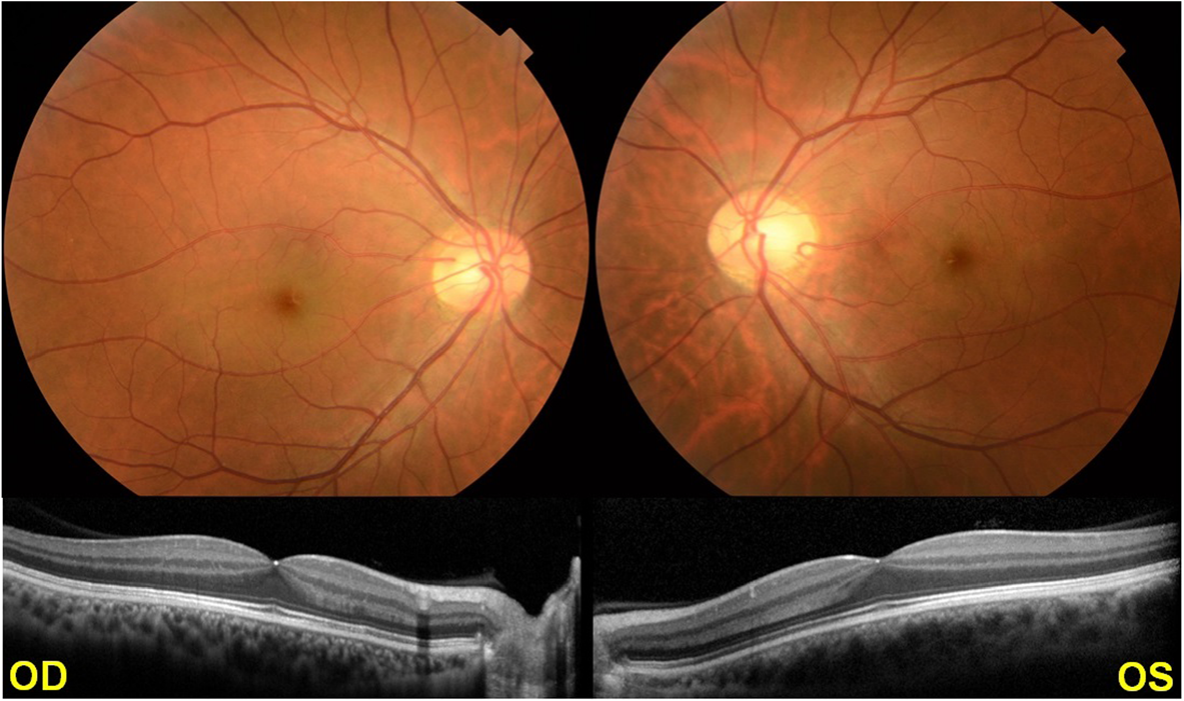
Color fundus photographs and optical coherence tomographic (OCT) images. Fundus photographs do not show any obvious abnormal findings, and OCT shows partial loss of the interdigitation zone

**Fig. 2:**
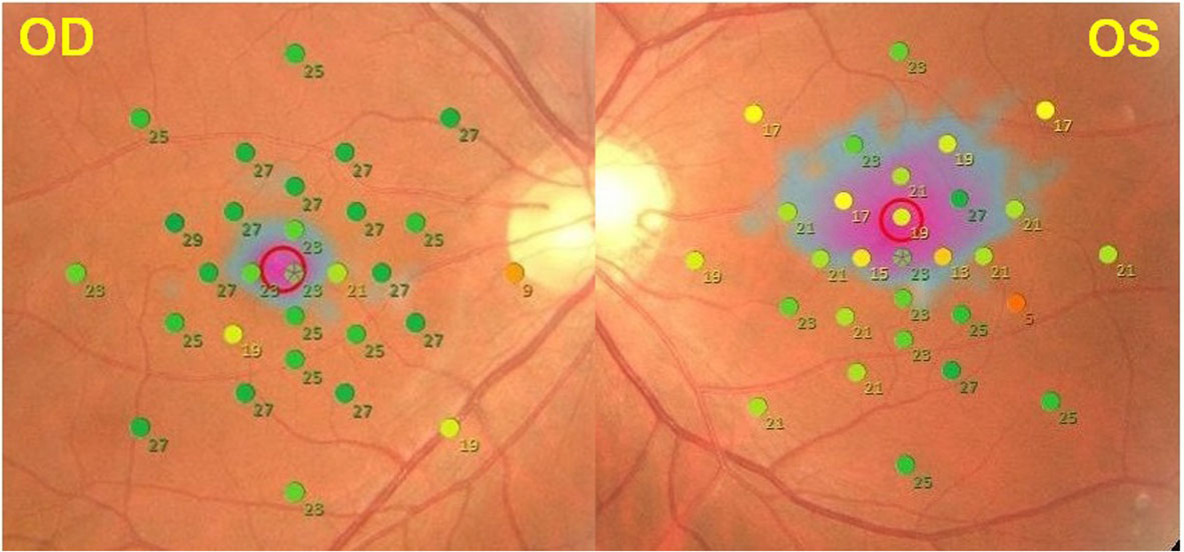
Retinal sensitivity measured by MP-3 microperimetry. Retinal sensitivity around fovea is decreased of both eyes

A 34-year-old man had reduced vision in his left eye for about 15 years, and night blindness for about two years. He had a complete ophthalmic examination including measurements of the best-corrected visual acuity (BCVA) and intraocular pressure, slit-lamp examinations, indirect ophthalmoscopy, Goldmann perimetry (GP), perimetry with Humphrey Field Analyzer (HFA), optical coherence tomography (OCT), MP-3 microperimetry, and full-field ERGs. His visual acuity was 20/15 in the right eye and 20/50 in the left eye. The fundus did not show any obvious abnormalities, but optical coherence tomography showed partial loss of the interdigitation zone (Fig. 1). The retinal sensitivity of the macular region was reduced in both eyes (Fig. 2). His family ocular histories were unknown because both his parents and grandparents were dead.

The ERGs were recorded and analyzed with MEB-9402 (NIHON KOHDEN, Tokyo, Japan) and LS-100 (Mayo, Nagoya, Japan) as light emitting device. Before ERG recordings, we confirmed that the pupils were maximally dilated to approximately 8.0 mm following topical application of a mixture of 0.5% tropicamide and 0.5% phenylephrine (Sandol P; Nitten Pharmaceutical Co. Ltd., Aichi, Japan). ERG recordings performed binocularly with contact lens electrodes.

He was dark-adapted for 20 min, and the dark-adapted 0.01 ERG, dark-adapted 3.0 ERG, and dark-adapted oscillatory potentials were recorded in that order. The eye was then light-adapted for 10 min, and the light-adapted 3.0 ERG, light-adapted 30 Hz flicker (3.0 photopic cd‧s‧m^− 2^ stimulus luminance) were recorded. Then, ERGs elicited by long-duration stimuli (on and off responses: 63.0 cd/m^2^ stimulus intensity with 31.6 cd/m^2^ background intensity, 200 ms duration) were recorded. All of the recordings conformed to the International Society for Clinical Electrophysiology of Vision (ISCEV) standard for full-field clinical electroretinography [2].

**Fig. 3:**
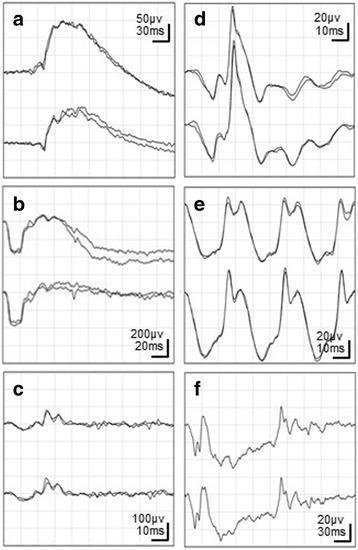
Electroretinogram (ERG) waveforms. **a** Dark-adapted 0.01; **b** dark-adapted 3.0; **c** dark-adapted oscillatory potentials; **d** light-adapted 3.0 ERG; **e** light-adapted 30 Hz flicker; **f** long-duration flashes (on-off responses). The upper line shows the result of the right eye and the lower line shows the result of the left eye. The recording of the on-off response was interrupted when the patient developed tonic seizure

During the recording of the dark-adapted ERG series elicited by single flashes, there were no changes in the patient’s overall condition. However, as soon as the flicker ERG recordings were completed and long-duration flashes began, the patient reported that a paralysis had developed in both upper limbs. Therefore, the examiner immediately stopped the ERG recordings, and soon thereafter, convulsions of the lower limbs developed and he lost consciousness. The convulsions disappeared after an intravenous injection of diazepam. Examinations of the ERG recordings, no apparent decrease in amplitudes or extension of implicit times were observed as compared with the normal waveforms indicated by ISCEV. No abnormality of the a-wave / b-wave ratio at the maximum combined response was observed (Fig. 3). Computed tomography of the head was performed on the same day, and no abnormality was found. Magnetic resonance imaging (MRI) of the head was also performed later, and the findings were normal. There was no abnormality in the electroencephalogram examination performed after the attack. After that incident, he reported that he had had conscious-loss seizures four years earlier.

## Discussion and conclusions

It has been known for more than a century that flickering artificial lighting and sunlight can induce epileptic seizures. It was already reported in 1934 that the electroencephalogram can be changed by photic stimulation [3]. Our patient was diagnosed with a photosensitive epileptic seizure because there were no abnormalities in the head MRI and no signs of partial seizure.

Many investigations have examined the epidemiology of photosensitivity seizures where photosensitivity was defined as a paroxysmal reaction to intermittent photic stimulation (IPS). These electroencephalographic responses are called photo paroxysmal response (PPR). A significant age dependency of PPR has been reported to be between ages 5 and 20 years [4]. Harding et al. reported that photosensitive epilepsy has a prevalence of approximately 1 in 4000 in the general population [5]. They also reported that the prevalence of women with PPR traits was up to 2.5 times higher than in men, and there was no difference in the prevalence of PPR by ethnic origins. Furthermore, the incidence of PPR was relatively high at 8.9% for normal children [6] and 0.5% for normal men [7]. Only 25% of patients lose their photosensitivity in their 20s and 30s, and the photosensitivity in the other patients persists in later life [8]. Therefore, it is necessary to pay attention to the risk of developing photosensitive epilepsy even if the subject is not a young woman as was our case.

Detailed investigations have also been made on the types of stimuli that can induce photosensitive epileptic seizures. Photosensitive seizures can be induced by a range of frequencies with a range of 10 to 30 Hz [9]. Most patients are sensitive to 16 Hz, 49% are sensitive to 50 Hz, and 15% are sensitive to 60 Hz [10]. About 3% of the light-sensitive population have systemic degenerative diseases and are sensitive to IPS of 1 to 3 Hz [11]. In our case, the seizure attack developed when the flicker stimulation ceased, and the on-off response stimulus was beginning to be presented. Thus, we believe that the light stimuli used in the flicker stimulation or the on-off response stimulation was the factor that induced the seizure. The frequency of flicker used in our case was 30 Hz, and the stimulation for the on-off responses was about 2.5 Hz and both used white light. Considering the results of previous studies, it is highly probable that the convulsions were induced by 30 Hz flicker, however the possibility of induction by the stimulation of on-off response cannot be completely be eliminated.

The wavelength of the stimulation light is also related to photosensitivity. Long wavelength red light has been reported to be more provocative even at low luminances [12]. Red light stimulation is sometimes used when investigating the functions of retinal ganglion cells [13, 14], thus attention is required in those cases.

It has been reported that stimulating only the fellow eye with shielding of one eye decreases the incidence of illusion and light sensitive seizures [15]. Therefore, it is assumed that photosensitivity seizures are less likely to occur with VEP, multifocal ERG, and the RETeval system recordings in which the stimuli are presented monocularly. This is in contrast to full-field ERG in which the stimuli are presented binocularly.

Harding et al. also reported that photosensitivity has a strong genetic tendency [8]. Twenty-five percent of mothers with photosensitivity show photosensitivity in the laboratory, and half of them develop photosensitive epileptic seizures. Therefore, it is recommended to confirm not only the patient’s past seizure episodes but also family history of seizures before the ERG recording.

In conclusion, there is a possibility of inducing a convulsive seizure with 30 Hz flicker or on-off stimulation. We recommend that clinicians inquire about past seizure episodes of the patients and their family before beginning the ERG recordings, and taking measures such as not conducting flicker or on-off response recording if there are any histories of seizures.

## Abbreviations

BCVA: Best corrected visual acuity
ERG: Electroretinogram
GP: Goldmann perimetry
HFA: Humphrey field analyzer
IPS: Intermittent photic stimulation
ISCEV: International Society for Clinical Electrophysiology of Vision
MRI: Magnetic resonance imaging
PPR: Photo paroxysmal response
VEP: Visual evoked potential
